# Whole-Genome Characterization of a Novel Human Influenza A(H1N2) Virus Variant, Brazil

**DOI:** 10.3201/eid2301.161122

**Published:** 2017-01

**Authors:** Paola Cristina Resende, Priscila Silva Born, Aline Rocha Matos, Fernando Couto Motta, Braulia Costa Caetano, Maria do Carmo Debur, Irina Nastassja Riediger, David Brown, Marilda M. Siqueira

**Affiliations:** Oswaldo Cruz Institute, Rio de Janeiro, Brazil (P.C. Resende, P.S. Born, A.R. Matos, F.C. Motta, B.C. Caetano, D. Brown, M.M. Siqueira);; Central Laboratory of the State of Parana, Curitiba, Brazil (M.C. Debur, I.N. Riediger)

**Keywords:** influenza A virus, viruses, influenza, H1N2v, subtypes, whole-genome characterization, reassortment, Brazil

## Abstract

We report the characterization of a novel reassortant influenza A(H1N2) virus not previously reported in humans. Recovered from a a pig farm worker in southeast Brazil who had influenza-like illness, this virus is a triple reassortant containing gene segments from subtypes H1N2 (hemagglutinin), H3N2 (neuraminidase), and pandemic H1N1 (remaining genes).

Influenza A(H1N2) viruses have been described in human, avian, and especially swine populations over many years (*1**,**2*). In contrast to the widespread circulation of seasonal H1N1 and H3N2 viruses, subtype H1N2 has been observed only sporadically in humans (*1**,**3**–**7*). Human H1N2 infections were reported during 1988–89 from sporadic cases over the winter in China (*3*). In 2000, another H1N2 subtype strain emerged in the human population and became widespread in Europe, with sporadic cases reported in the Middle East, Asia, Africa, and the Americas during 2001–2003 (*1**,**4*). In Brazil, this H1N2 subtype strain was detected in humans in the southeast region during the winter of 2002 and in the northern region at the beginning of 2003 (*5*). This 2000–2003 H1N2 subtype strain had a genetic origin similar to the 1988–1989 H1N2 strain from China, both reassortants between human seasonal H1N1 and H3N2 subtype lineages (*3**,**4*). 

In contrast, sporadic cases of zoonotic human infections with swine-origin H1N2 subtype variants (H1N2v) have also been described (*6**,**7*). In Brazil, the passive monitoring of influenza A viruses in pigs has taken place since 2009 (*8*). Recently, a phylogenetic study revealed that H1N2 subtype viruses have circulated undetected in swine herds in Brazil for more than a decade, and reassortments may have occurred (*9*). These viruses seem to be reassortants originating from an ancestor virus introduced to pigs from humans in the late 1990s and early 2000s and remained as a relic from a now-extinct human-host hemagglutinin lineage. However, after the emergence in humans of influenza A(H1N1)pdm09 in 2009, reassortment events lead to H1N2 viruses acquiring internal genes segments from the pandemic strain (*9*). 

Even though these H1N2 subtype strains from Brazil circulating in the swine population, they have not been detected in humans. We report detection and characterization of a variant H1N2 subtype strain (H1N2v) with a genomic origin not previously reported in humans.

This virus was identified from a nasopharyngeal aspirate collected on November 26, 2015, from a 16-year-old girl from a rural area in Castro City, Paraná, in the southern region of Brazil. Castro has ≈67,000 inhabitants and is a major livestock hub for dairy cattle, poultry, and pigs. The patient did not present any risk factors for influenza but showed development of an influenza-like illness with an onset of symptoms (fever, cough, sore throat, chest pain, and myalgia) on November 23, 2015. The follow-up local investigation reported that she had been working at a swine farm and confirmed direct patient contact with pigs. She had not received a prior influenza vaccine or antiviral treatment, and her clinical recovery was uneventful. 

The virus sample was sent to the Central Laboratory of the State of Parana, where an influenza A virus strain was detected. This strain could not be further subtyped using the influenza real-time reverse transcription PCR protocol recommended by the World Health Organization. 

Genomic Sanger sequencing of the strain was conducted at the National Influenza Center, IOC, FIOCRUZ, Rio de Janeiro, Brazil. BLAST (https://blast.ncbi.nlm.nih.gov/Blast.cgi) was performed for each gene segment sequenced and revealed strong identity with an H1N2 virus subtype genome detected in swine in Santa Catarina, a state in southern Brazil, in 2011 (Table). The human viruses with closest identity to the H1N2v virus detected in this study were a 2003 H1N2 subtype human lineage (hemagglutinin gene), a 1998 H3N2 subtype human seasonal lineage (neuraminidase gene), and A(H1N1)pdm09 subtype lineage for all other genes. This virus isolate was designated as influenza A/Parana/720/2015(H1N2v); the genome sequence is available in the GISAID database (http://platform.gisaid.org; submission no. EPI_ISL_223342). 

**Table:** Highest degree of gene identity of the influenza A/Parana/720/2015 (H1N2)v subtype strain identified from a patient in Brazil with other swine and human strains*

Gene segment	BLAST hits†	% Identity
PB2	A/swine/Brazil/185-11-7/2011(H1N2)	98
	A/San Diego/INS194/2009(H1N1)	98
PB1	A/swine/Brazil/185-11-7/2011(H1N2)	99
	A/Singapore/GN285/2009(H1N1)	98
PA	A/swine/Brazil/185-11-7/2011(H1N2)	98
	A/Texas/67/2009(H1N1)	98
HA	A/swine/Brazil/185-11-7/2011(H1N2)	97
	A/New York/487/2003(H1N2)	95
NP	A/swine/Brazil/185-11-7/2011(H1N2)	99
	A/Tennessee/F1057c56/2010(H1N1)	98
NA	A/swine/Brazil/185-11-7/2011(H1N2)	97
	A/Malaysia/17392/1998(H3N2)	93
M	A/swine/Brazil/185-11-7/2011(H1N2)	99
	A/Mexico City/INER16/2009(H1N1)	99
NS	A/swine/Brazil/185-11-7/2011(H1N2)	98
	A/Singapore/TT454/2010(H1N1)	98

*Sequence submitted to GISAID (http://platform.gisaid.org; submission no. EPI_ISL_223342). HA, hemagglutinin gene; M, matrix gene; NA, neuraminidase gene, NP, nucleoprotein gene; NS, nonstructural protein gene; PB2, polymerase basic 2 gene; PB1, polymerase basic gene; PA, polymerase acid gene. †Sequence with major identity on BLAST.

We conducted phylogenetic reconstructions of each gene segment by the maximum-likelihood method using a dataset with all H1N2 subtype sequences and some representative sequences for H1N1 and H3N2 subtypes available in influenza genetic databases (Technical Appendix). Our analysis revealed that each gene segment of A/Parana/720/2015(H1N2v) had the same phylogenetic profile as recent swine H1N2 subtype sequences from Brazil. This supports the BLAST findings and suggests a recent swine–human infection by the H1N2v strain. Because similar swine strains have been identified in pigs ≈300 km distant from where the human case occurred (*9*), the virus is likely to have been circulating in pigs in Castro City. The H1N2v subtype we report contained the S31N marker in the matrix 2 protein, which confers resistance to the adamantane antiviral class, similar to A(H1N1)pdm09 viruses (*10*). 

To date, no further H1N2 subtype human cases have been detected in Brazil; however, influenza virus strains from this region and period are under investigation to confirm whether more H1N2v subtype cases may have occurred. This report highlights the need for influenza surveillance in humans and animals, as well as in their interface, especially during influenza season when transmission is high. To ensure early detection, surveillance should focus on geographic areas when influenza A viruses subtypes co-circulate and where human–animal contact is frequent.

Technical Appendix Maximum-likelihood trees of each gene segment used to reconstruct the phylogenetic profile of influenza A/Parana/720/2015 (H1N2)v.

## References

[1] Komadina N, McVernon J, Hall R, Leder K. A historical perspective of influenza A(H1N2) virus. Emerg Infect Dis. 2014;20:6–12. 10.3201/eid2001.121848 24377419 PMC3884707

[2] Lorusso A, Vincent AL, Gramer MR, Lager KM, Ciacci-Zanella JR. Contemporary epidemiology of North American lineage triple reassortant influenza A viruses in pigs. In: Richt AJ, Webby JR, editors. Swine influenza. Berlin: Springer Berlin Heidelberg; 2013. p. 113–31. 10.1007/82_2011_196 PMC7120137 22266673

[3] Guo YJ, Xu XY, Cox NJ. Human influenza A (H1N2) viruses isolated from China. J Gen Virol. 1992;73:383–7. 10.1099/0022-1317-73-2-383 1538194

[4] Chen MJ, La T, Zhao P, Tam JS, Rappaport R, Cheng SM. Genetic and phylogenetic analysis of multi-continent human influenza A(H1N2) reassortant viruses isolated in 2001 through 2003. Virus Res. 2006;122:200–5. 10.1016/j.virusres.2006.07.010 16971014

[5] de Mattos Silva Oliveira TF, Yokosawa J, Motta FC, Siqueira MM, da Silveira HL, Queiróz DA. Molecular characterization of influenza viruses collected from young children in Uberlandia, Brazil - from 2001 to 2010. BMC Infect Dis. 2015;15:71. 10.1186/s12879-015-0817-z 25886886 PMC4336712

[6] Shinde V, Bridges CB, Uyeki TM, Shu B, Balish A, Xu X, et al. Triple-reassortant swine influenza A (H1) in humans in the United States, 2005-2009. N Engl J Med. 2009;360:2616–25. 10.1056/NEJMoa0903812 19423871

[7] Centers for Disease Control and Prevention. Influenza (FLU). H1N2 variant virus detected in Minnesota [cited 2016 Jun 1]. http://www.cdc.gov/flu/spotlights/h1n2v-cases-mn.htm

[8] Schaefer R, Rech RR, Gava D, Cantão ME, da Silva MC, Silveira S, et al. A human-like H1N2 influenza virus detected during an outbreak of acute respiratory disease in swine in Brazil. Arch Virol. 2015;160:29–38. 10.1007/s00705-014-2223-z 25209152

[9] Nelson MI, Schaefer R, Gava D, Cantão ME, Ciacci-Zanella JR. Emerg Infect Dis. 2015;21:1339–47. 10.3201/eid2108.141891 26196759 PMC4517702

[10] Dawood FS, Jain S, Finelli L, Shaw MW, Lindstrom S, Garten RJ, et al.; Novel Swine-Origin Influenza A (H1N1) Virus Investigation Team. Emergence of a novel swine-origin influenza A (H1N1) virus in humans. N Engl J Med. 2009;360:2605–15. 10.1056/NEJMoa0903810 19423869

