# Supplementary material for: Whole-Genome Characterization of a Novel Human Influenza A(H1N2) Virus Variant, Brazil
Source: Emerg Infect Dis. 2017 Jan;23(1):152–4. doi: 10.3201/eid2301.161122 (PMC5176240; doi:10.3201/eid2301.161122)
Supplement: Technical Appendix — Maximum-likelihood trees of each gene segment used to reconstruct the phylogenetic profile of influenza A/Parana/720/2015 (H1N2)v. [file 16-1122-Techapp-s1.pdf]

Article DOI: http://dx.doi.org/10.3201/eid2301.161122

Whole-Genome Characterization of a Novel Human Influenza A(H1N2) Virus Variant, Brazil

Technical Appendix

Technical Appendix Figure (following pages). Maximum-likelihood (ML) trees of each gene segment (PB2, polymerase basic 2 gene; PB1, polymerase basic gene; PA, polymerase acid gene; HA, hemagglutinin gene; NP, nucleoprotein gene; NA, neuraminidase gene, M, matrix gene; NS, non-structural protein gene) were performed to reconstruct the phylogenetic profile of influenza A/Parana/720/2015 (H1N2)v (Figure, red). The dataset used includes H1N1 (orange) and H3N2 (blue) seasonal and H1N1pdm09 (pink) vaccine strains and all H1N2 whole genomes available in GenBank or the Global Initiative on Sharing All Influenza Data. The H1N2 subtype strains were classified as Brazilian swine H1N2 (green), worldwide swine and avian (black), human H1N2 and swine-origin H1N2v strains (purple). The Hasegawa-Kishino-Yano + gamma distribution among the sites was the best-fitting nucleotide substitution model and was used to reconstruct the trees. ML reliability of branches was evaluated using an approximate likelihood ratio test; the interior support branch cutoff value was ≥0.9.

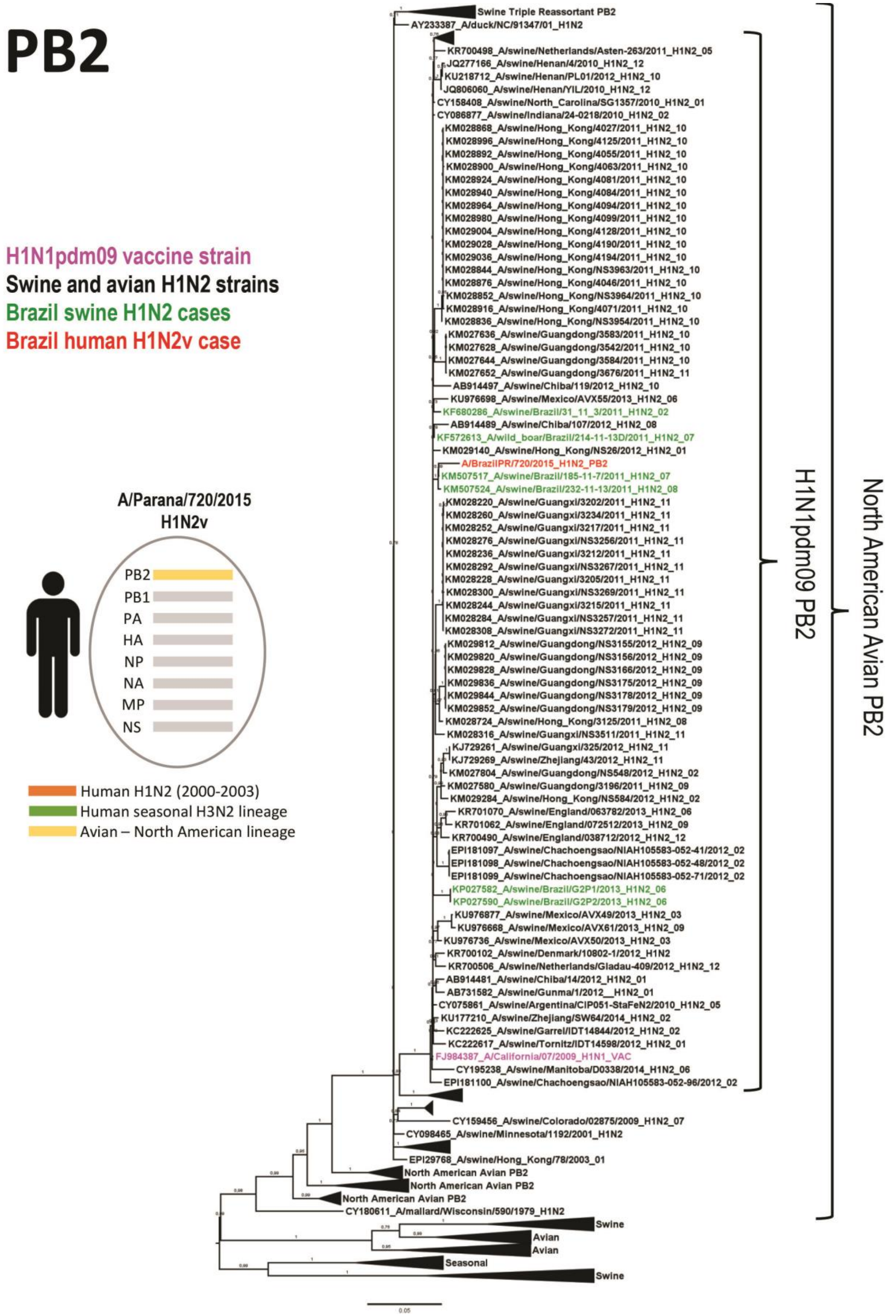
PB2
H1N1pdm09 vaccine strain
Swine and avian H1N2 strains
Brazil swine H1N2 cases
Brazil human H1N2v case
A/Parana/720/2015
H1N2v
PB2
PB1
PA
HA
NP
NA
MP
NS
Human H1N2 (2000-2003)
Human seasonal H3N2 lineage
Avian – North American lineage
Swine Triple Reassortant PB2
AY233387_A/duck/NC/91347/01_H1N2
KR700498_A/swine/Netherlands/Asten-263/2011_H1N2_05
JQ277166_A/swine/Henan/4/2010_H1N2_12
KU218712_A/swine/Henan/PL01/2012_H1N2_10
JQ806060_A/swine/Henan/YIL/2010_H1N2_12
CY158408_A/swine/North_Carolina/SG1357/2010_H1N2_01
CY086877_A/swine/Indiana/24-0218/2010_H1N2_02
KM028868_A/swine/Hong_Kong/4027/2011_H1N2_10
KM028996_A/swine/Hong_Kong/4125/2011_H1N2_10
KM028892_A/swine/Hong_Kong/4055/2011_H1N2_10
KM028900_A/swine/Hong_Kong/4063/2011_H1N2_10
KM028924_A/swine/Hong_Kong/4081/2011_H1N2_10
KM028940_A/swine/Hong_Kong/4084/2011_H1N2_10
KM028964_A/swine/Hong_Kong/4094/2011_H1N2_10
KM028980_A/swine/Hong_Kong/4099/2011_H1N2_10
KM029004_A/swine/Hong_Kong/4128/2011_H1N2_10
KM029028_A/swine/Hong_Kong/4190/2011_H1N2_10
KM029036_A/swine/Hong_Kong/4194/2011_H1N2_10
KM028844_A/swine/Hong_Kong/NS3963/2011_H1N2_10
KM028876_A/swine/Hong_Kong/4046/2011_H1N2_10
KM028852_A/swine/Hong_Kong/NS3964/2011_H1N2_10
KM028916_A/swine/Hong_Kong/4071/2011_H1N2_10
KM028836_A/swine/Hong_Kong/NS3954/2011_H1N2_10
KM027636_A/swine/Guangdong/3583/2011_H1N2_10
KM027628_A/swine/Guangdong/3542/2011_H1N2_10
KM027644_A/swine/Guangdong/3584/2011_H1N2_10
KM027652_A/swine/Guangdong/3676/2011_H1N2_11
AB914497_A/swine/Chiba/119/2012_H1N2_10
KU976698_A/swine/Mexico/AVX55/2013_H1N2_06
KF680286_A/swine/Brazil/31_11_3/2011_H1N2_02
AB914489_A/swine/Chiba/107/2012_H1N2_08
KF572613_A/wild_boar/Brazil/214-11-13D/2011_H1N2_07
KM029140_A/swine/Hong_Kong/NS26/2012_H1N2_01
A/BrazilPR/720/2015_H1N2_PB2
KM507517_A/swine/Brazil/185-11-7/2011_H1N2_07
KM507524_A/swine/Brazil/232-11-13/2011_H1N2_08
KM028220_A/swine/Guangxi/3202/2011_H1N2_11
KM028260_A/swine/Guangxi/3234/2011_H1N2_11
KM028252_A/swine/Guangxi/3217/2011_H1N2_11
KM028276_A/swine/Guangxi/NS3256/2011_H1N2_11
KM028236_A/swine/Guangxi/3212/2011_H1N2_11
KM028292_A/swine/Guangxi/NS3267/2011_H1N2_11
KM028228_A/swine/Guangxi/3205/2011_H1N2_11
KM028300_A/swine/Guangxi/NS3269/2011_H1N2_11
KM028244_A/swine/Guangxi/3215/2011_H1N2_11
KM028284_A/swine/Guangxi/NS3257/2011_H1N2_11
KM028308_A/swine/Guangxi/NS3272/2011_H1N2_11
KM029812_A/swine/Guangdong/NS3155/2012_H1N2_09
KM029820_A/swine/Guangdong/NS3156/2012_H1N2_09
KM029828_A/swine/Guangdong/NS3166/2012_H1N2_09
KM029836_A/swine/Guangdong/NS3175/2012_H1N2_09
KM029844_A/swine/Guangdong/NS3178/2012_H1N2_09
KM029852_A/swine/Guangdong/NS3179/2012_H1N2_09
KM028724_A/swine/Hong_Kong/3125/2011_H1N2_08
KM028316_A/swine/Guangxi/NS3511/2011_H1N2_11
KJ729261_A/swine/Guangxi/325/2012_H1N2_11
KJ729269_A/swine/Zhejiang/43/2012_H1N2_11
KM027804_A/swine/Guangdong/NS548/2012_H1N2_02
KM027580_A/swine/Guangdong/3196/2011_H1N2_09
KM029284_A/swine/Hong_Kong/NS584/2012_H1N2_02
KR701070_A/swine/England/063782/2013_H1N2_06
KR701062_A/swine/England/072512/2013_H1N2_09
KR700490_A/swine/England/038712/2012_H1N2_12
EPI181097_A/swine/Chachoengsao/NIAH105583-052-41/2012_02
EPI181098_A/swine/Chachoengsao/NIAH105583-052-48/2012_02
EPI181099_A/swine/Chachoengsao/NIAH105583-052-71/2012_02
KP027582_A/swine/Brazil/G2P1/2013_H1N2_06
KP027590_A/swine/Brazil/G2P2/2013_H1N2_06
KU976877_A/swine/Mexico/AVX49/2013_H1N2_03
KU976668_A/swine/Mexico/AVX61/2013_H1N2_09
KU976736_A/swine/Mexico/AVX50/2013_H1N2_03
KR700102_A/swine/Denmark/10802-1/2012_H1N2
KR700506_A/swine/Netherlands/Gladau-409/2012_H1N2_12
AB914481_A/swine/Chiba/14/2012_H1N2_01
AB731582_A/swine/Gunma/1/2012__H1N2_01
CY075861_A/swine/Argentina/CIP051-StaFeN2/2010_H1N2_05
KU177210_A/swine/Zhejiang/SW64/2014_H1N2_02
KC222625_A/swine/Garrel/IDT14844/2012_H1N2_02
KC222617_A/swine/Tornitz/IDT14598/2012_H1N2_01
FJ984387_A/California/07/2009_H1N1_VAC
CY195238_A/swine/Manitoba/D0338/2014_H1N2_06
EPI181100_A/swine/Chachoengsao/NIAH105583-052-96/2012_02
CY159456_A/swine/Colorado/02875/2009_H1N2_07
CY098465_A/swine/Minnesota/1192/2001_H1N2
EPI29768_A/swine/Hong_Kong/78/2003_01
North American Avian PB2
North American Avian PB2
North American Avian PB2
CY180611_A/mallard/Wisconsin/590/1979_H1N2
Swine
Avian
Avian
Seasonal
Swine
0.05
H1N1pdm09 PB2
North American Avian PB2

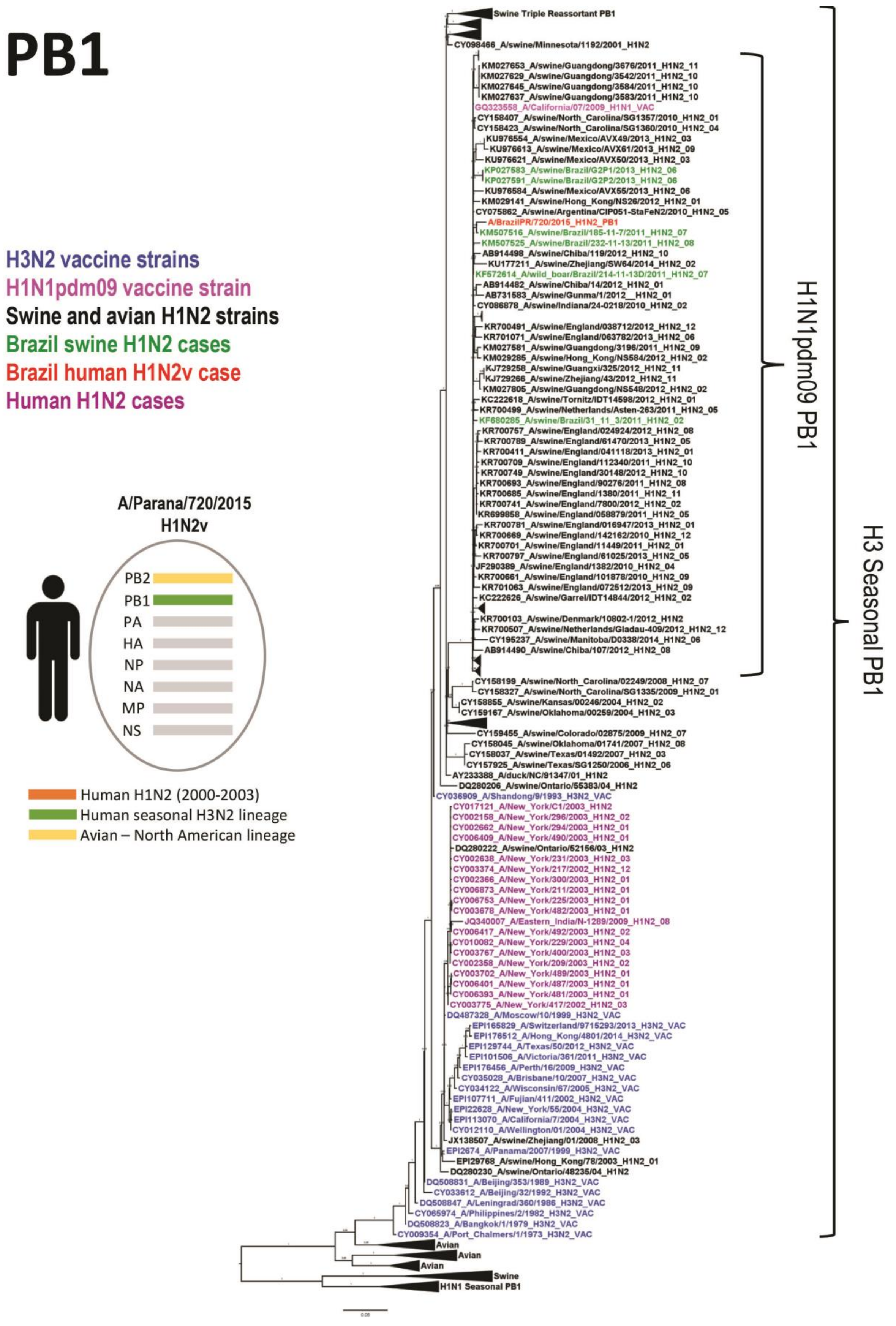
PB1
H3N2 vaccine strains
H1N1pdm09 vaccine strain
Swine and avian H1N2 strains
Brazil swine H1N2 cases
Brazil human H1N2v case
Human H1N2 cases
A/Parana/720/2015
H1N2v
PB2
PB1
PA
HA
NP
NA
MP
NS
Human H1N2 (2000-2003)
Human seasonal H3N2 lineage
Avian – North American lineage
Swine Triple Reassortant PB1
CY098466_A/swine/Minnesota/1192/2001_H1N2
KM027653_A/swine/Guangdong/3676/2011_H1N2_11
KM027629_A/swine/Guangdong/3542/2011_H1N2_10
KM027645_A/swine/Guangdong/3584/2011_H1N2_10
KM027637_A/swine/Guangdong/3583/2011_H1N2_10
GQ323558_A/California/07/2009_H1N1_VAC
CY158407_A/swine/North_Carolina/SG1357/2010_H1N2_01
CY158423_A/swine/North_Carolina/SG1360/2010_H1N2_04
KU976554_A/swine/Mexico/AVX49/2013_H1N2_03
KU976613_A/swine/Mexico/AVX61/2013_H1N2_09
KU976621_A/swine/Mexico/AVX50/2013_H1N2_03
KP027583_A/swine/Brazil/G2P1/2013_H1N2_06
KP027591_A/swine/Brazil/G2P2/2013_H1N2_06
KU976584_A/swine/Mexico/AVX55/2013_H1N2_06
KM029141_A/swine/Hong_Kong/NS26/2012_H1N2_01
CY075862_A/swine/Argentina/CIP051-StaFeN2/2010_H1N2_05
A/BrazilPR/720/2015_H1N2_PB1
KM507516_A/swine/Brazil/185-11-7/2011_H1N2_07
KM507525_A/swine/Brazil/232-11-13/2011_H1N2_08
AB914498_A/swine/Chiba/119/2012_H1N2_10
KU177211_A/swine/Zhejiang/SW64/2014_H1N2_02
KF572614_A/wild_boar/Brazil/214-11-13D/2011_H1N2_07
AB914482_A/swine/Chiba/14/2012_H1N2_01
AB731583_A/swine/Gunma/1/2012__H1N2_01
CY086878_A/swine/Indiana/24-0218/2010_H1N2_02
KR700491_A/swine/England/038712/2012_H1N2_12
KR701071_A/swine/England/063782/2013_H1N2_06
KM027581_A/swine/Guangdong/3196/2011_H1N2_09
KM029285_A/swine/Hong_Kong/NS584/2012_H1N2_02
KJ729258_A/swine/Guangxi/325/2012_H1N2_11
KJ729266_A/swine/Zhejiang/43/2012_H1N2_11
KM027805_A/swine/Guangdong/NS548/2012_H1N2_02
KC222618_A/swine/Tornitz/IDT14598/2012_H1N2_01
KR700499_A/swine/Netherlands/Asten-263/2011_H1N2_05
KF680285_A/swine/Brazil/31_11_3/2011_H1N2_02
KR700757_A/swine/England/024924/2012_H1N2_08
KR700789_A/swine/England/61470/2013_H1N2_05
KR700411_A/swine/England/041118/2013_H1N2_01
KR700709_A/swine/England/112340/2011_H1N2_10
KR700749_A/swine/England/30148/2012_H1N2_10
KR700693_A/swine/England/90276/2011_H1N2_08
KR700685_A/swine/England/1380/2011_H1N2_11
KR700741_A/swine/England/7800/2012_H1N2_02
KR699858_A/swine/England/058879/2011_H1N2_05
KR700781_A/swine/England/016947/2013_H1N2_01
KR700669_A/swine/England/142162/2010_H1N2_12
KR700701_A/swine/England/11449/2011_H1N2_01
KR700797_A/swine/England/61025/2013_H1N2_05
JF290389_A/swine/England/1382/2010_H1N2_04
KR700661_A/swine/England/101878/2010_H1N2_09
KR701063_A/swine/England/072512/2013_H1N2_09
KC222626_A/swine/Garrel/IDT14844/2012_H1N2_02
KR700103_A/swine/Denmark/10802-1/2012_H1N2
KR700507_A/swine/Netherlands/Gladau-409/2012_H1N2_12
CY195237_A/swine/Manitoba/D0338/2014_H1N2_06
AB914490_A/swine/Chiba/107/2012_H1N2_08
CY158199_A/swine/North_Carolina/02249/2008_H1N2_07
CY158327_A/swine/North_Carolina/SG1335/2009_H1N2_01
CY158855_A/swine/Kansas/00246/2004_H1N2_02
CY159167_A/swine/Oklahoma/00259/2004_H1N2_03
CY159455_A/swine/Colorado/02875/2009_H1N2_07
CY158045_A/swine/Oklahoma/01741/2007_H1N2_08
CY158037_A/swine/Texas/01492/2007_H1N2_03
CY157925_A/swine/Texas/SG1250/2006_H1N2_06
AY233388_A/duck/NC/91347/01_H1N2
DQ280206_A/swine/Ontario/55383/04_H1N2
CY036909_A/Shandong/9/1993_H3N2_VAC
CY017121_A/New_York/C1/2003_H1N2
CY002158_A/New_York/296/2003_H1N2_02
CY002662_A/New_York/294/2003_H1N2_01
CY006409_A/New_York/490/2003_H1N2_01
DQ280222_A/swine/Ontario/52156/03_H1N2
CY002638_A/New_York/231/2003_H1N2_03
CY003374_A/New_York/217/2002_H1N2_12
CY002366_A/New_York/300/2003_H1N2_01
CY006873_A/New_York/211/2003_H1N2_01
CY006753_A/New_York/225/2003_H1N2_01
CY003678_A/New_York/482/2003_H1N2_01
JQ340007_A/Eastern_India/N-1289/2009_H1N2_08
CY006417_A/New_York/492/2003_H1N2_02
CY010082_A/New_York/229/2003_H1N2_04
CY003767_A/New_York/400/2003_H1N2_03
CY002358_A/New_York/209/2003_H1N2_02
CY003702_A/New_York/489/2003_H1N2_01
CY006401_A/New_York/487/2003_H1N2_01
CY006393_A/New_York/481/2003_H1N2_01
CY003775_A/New_York/417/2002_H1N2_03
DQ487328_A/Moscow/10/1999_H3N2_VAC
EPI165829_A/Switzerland/9715293/2013_H3N2_VAC
EPI176512_A/Hong_Kong/4801/2014_H3N2_VAC
EPI129744_A/Texas/50/2012_H3N2_VAC
EPI101506_A/Victoria/361/2011_H3N2_VAC
EPI176456_A/Perth/16/2009_H3N2_VAC
CY035028_A/Brisbane/10/2007_H3N2_VAC
CY034122_A/Wisconsin/67/2005_H3N2_VAC
EPI107711_A/Fujian/411/2002_H3N2_VAC
EPI22628_A/New_York/55/2004_H3N2_VAC
EPI113070_A/California/7/2004_H3N2_VAC
CY012110_A/Wellington/01/2004_H3N2_VAC
JX138507_A/swine/Zhejiang/01/2008_H1N2_03
EPI2674_A/Panama/2007/1999_H3N2_VAC
EPI29768_A/swine/Hong_Kong/78/2003_H1N2_01
DQ280230_A/swine/Ontario/48235/04_H1N2
DQ508831_A/Beijing/353/1989_H3N2_VAC
CY033612_A/Beijing/32/1992_H3N2_VAC
DQ508847_A/Leningrad/360/1986_H3N2_VAC
CY065974_A/Philippines/2/1982_H3N2_VAC
DQ508823_A/Bangkok/1/1979_H3N2_VAC
CY009354_A/Port_Chalmers/1/1973_H3N2_VAC
Avian
Avian
Avian
Swine
H1N1 Seasonal PB1
H1N1pdm09 PB1
H3 Seasonal PB1

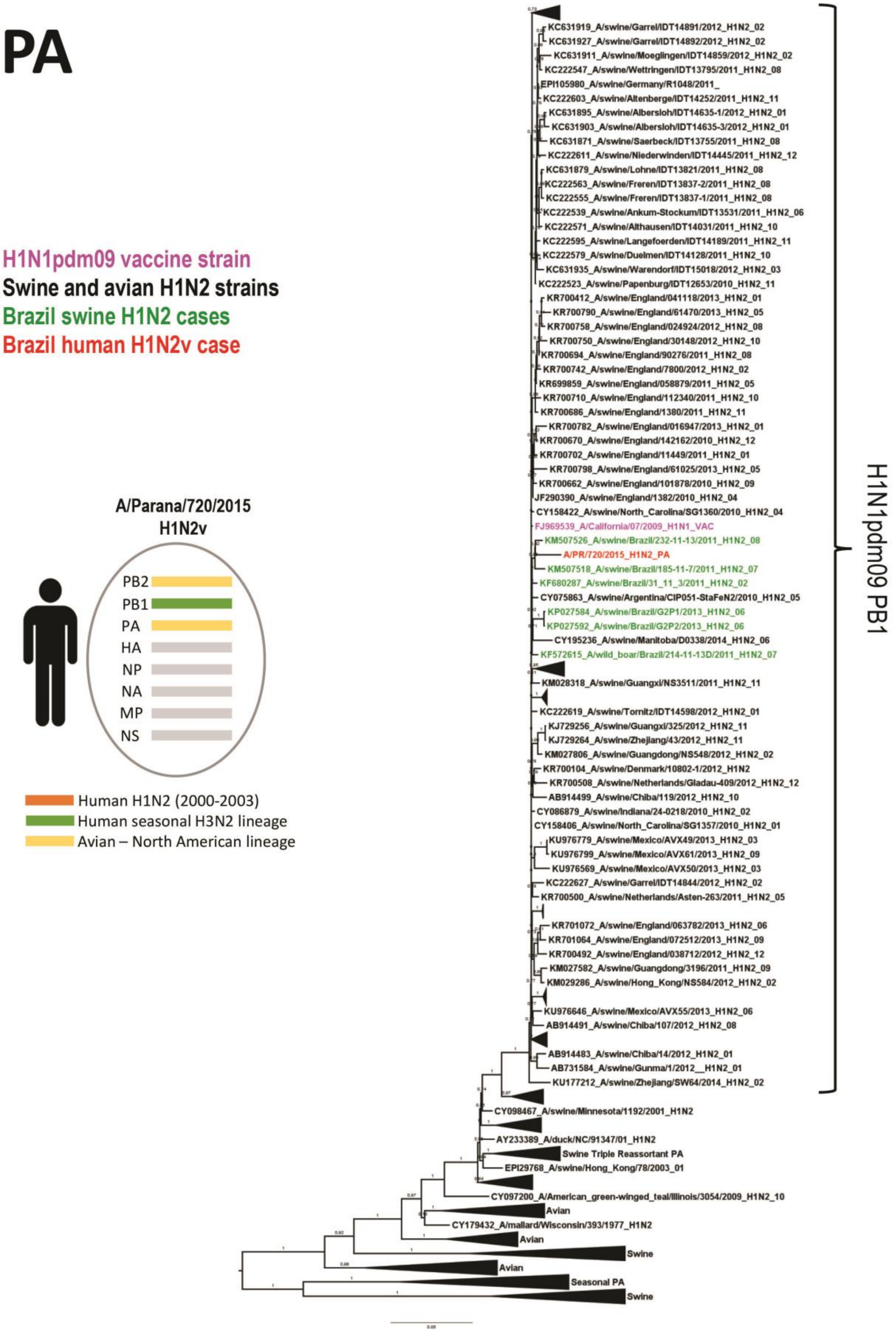
PA
H1N1pdm09 vaccine strain
Swine and avian H1N2 strains
Brazil swine H1N2 cases
Brazil human H1N2v case
A/Parana/720/2015
H1N2v
PB2
PB1
PA
HA
NP
NA
MP
NS
Human H1N2 (2000-2003)
Human seasonal H3N2 lineage
Avian – North American lineage
KC631919_A/swine/Garrel/IDT14891/2012_H1N2_02
KC631927_A/swine/Garrel/IDT14892/2012_H1N2_02
KC631911_A/swine/Moeglingen/IDT14859/2012_H1N2_02
KC222547_A/swine/Wettringen/IDT13795/2011_H1N2_08
EPI105980_A/swine/Germany/R1048/2011_
KC222603_A/swine/Altenberge/IDT14252/2011_H1N2_11
KC631895_A/swine/Albersloh/IDT14635-1/2012_H1N2_01
KC631903_A/swine/Albersloh/IDT14635-3/2012_H1N2_01
KC631871_A/swine/Saerbeck/IDT13755/2011_H1N2_08
KC222611_A/swine/Niederwinden/IDT14445/2011_H1N2_12
KC631879_A/swine/Lohne/IDT13821/2011_H1N2_08
KC222563_A/swine/Freren/IDT13837-2/2011_H1N2_08
KC222555_A/swine/Freren/IDT13837-1/2011_H1N2_08
KC222539_A/swine/Ankum-Stockum/IDT13531/2011_H1N2_06
KC222571_A/swine/Althausen/IDT14031/2011_H1N2_10
KC222595_A/swine/Langefoerden/IDT14189/2011_H1N2_11
KC222579_A/swine/Duelmen/IDT14128/2011_H1N2_10
KC631935_A/swine/Warendorf/IDT15018/2012_H1N2_03
KC222523_A/swine/Papenburg/IDT12653/2010_H1N2_11
KR700412_A/swine/England/041118/2013_H1N2_01
KR700790_A/swine/England/61470/2013_H1N2_05
KR700758_A/swine/England/024924/2012_H1N2_08
KR700750_A/swine/England/30148/2012_H1N2_10
KR700694_A/swine/England/90276/2011_H1N2_08
KR700742_A/swine/England/7800/2012_H1N2_02
KR699859_A/swine/England/058879/2011_H1N2_05
KR700710_A/swine/England/112340/2011_H1N2_10
KR700686_A/swine/England/1380/2011_H1N2_11
KR700782_A/swine/England/016947/2013_H1N2_01
KR700670_A/swine/England/142162/2010_H1N2_12
KR700702_A/swine/England/11449/2011_H1N2_01
KR700798_A/swine/England/61025/2013_H1N2_05
KR700662_A/swine/England/101878/2010_H1N2_09
JF290390_A/swine/England/1382/2010_H1N2_04
CY158422_A/swine/North_Carolina/SG1360/2010_H1N2_04
FJ969539_A/California/07/2009_H1N1_VAC
KM507526_A/swine/Brazil/232-11-13/2011_H1N2_08
A/PR/720/2015_H1N2_PA
KM507518_A/swine/Brazil/185-11-7/2011_H1N2_07
KF680287_A/swine/Brazil/31_11_3/2011_H1N2_02
CY075863_A/swine/Argentina/CIP051-StaFeN2/2010_H1N2_05
KP027584_A/swine/Brazil/G2P1/2013_H1N2_06
KP027592_A/swine/Brazil/G2P2/2013_H1N2_06
CY195236_A/swine/Manitoba/D0338/2014_H1N2_06
KF572615_A/wild_boar/Brazil/214-11-13D/2011_H1N2_07
KM028318_A/swine/Guangxi/NS3511/2011_H1N2_11
KC222619_A/swine/Tornitz/IDT14598/2012_H1N2_01
KJ729256_A/swine/Guangxi/325/2012_H1N2_11
KJ729264_A/swine/Zhejiang/43/2012_H1N2_11
KM027806_A/swine/Guangdong/NS548/2012_H1N2_02
KR700104_A/swine/Denmark/10802-1/2012_H1N2
KR700508_A/swine/Netherlands/Gladau-409/2012_H1N2_12
AB914499_A/swine/Chiba/119/2012_H1N2_10
CY086879_A/swine/Indiana/24-0218/2010_H1N2_02
CY158406_A/swine/North_Carolina/SG1357/2010_H1N2_01
KU976779_A/swine/Mexico/AVX49/2013_H1N2_03
KU976799_A/swine/Mexico/AVX61/2013_H1N2_09
KU976569_A/swine/Mexico/AVX50/2013_H1N2_03
KC222627_A/swine/Garrel/IDT14844/2012_H1N2_02
KR700500_A/swine/Netherlands/Asten-263/2011_H1N2_05
KR701072_A/swine/England/063782/2013_H1N2_06
KR701064_A/swine/England/072512/2013_H1N2_09
KR700492_A/swine/England/038712/2012_H1N2_12
KM027582_A/swine/Guangdong/3196/2011_H1N2_09
KM029286_A/swine/Hong_Kong/NS584/2012_H1N2_02
KU976646_A/swine/Mexico/AVX55/2013_H1N2_06
AB914491_A/swine/Chiba/107/2012_H1N2_08
AB914483_A/swine/Chiba/14/2012_H1N2_01
AB731584_A/swine/Gunma/1/2012__H1N2_01
KU177212_A/swine/Zhejiang/SW64/2014_H1N2_02
H1N1pdm09 PB1
CY098467_A/swine/Minnesota/1192/2001_H1N2
AY233389_A/duck/NC/91347/01_H1N2
Swine Triple Reassortant PA
EPI29768_A/swine/Hong_Kong/78/2003_01
CY097200_A/American_green-winged_teal/Illinois/3054/2009_H1N2_10
Avian
CY179432_A/mallard/Wisconsin/393/1977_H1N2
Avian
Swine
Avian
Seasonal PA
Swine
0.05

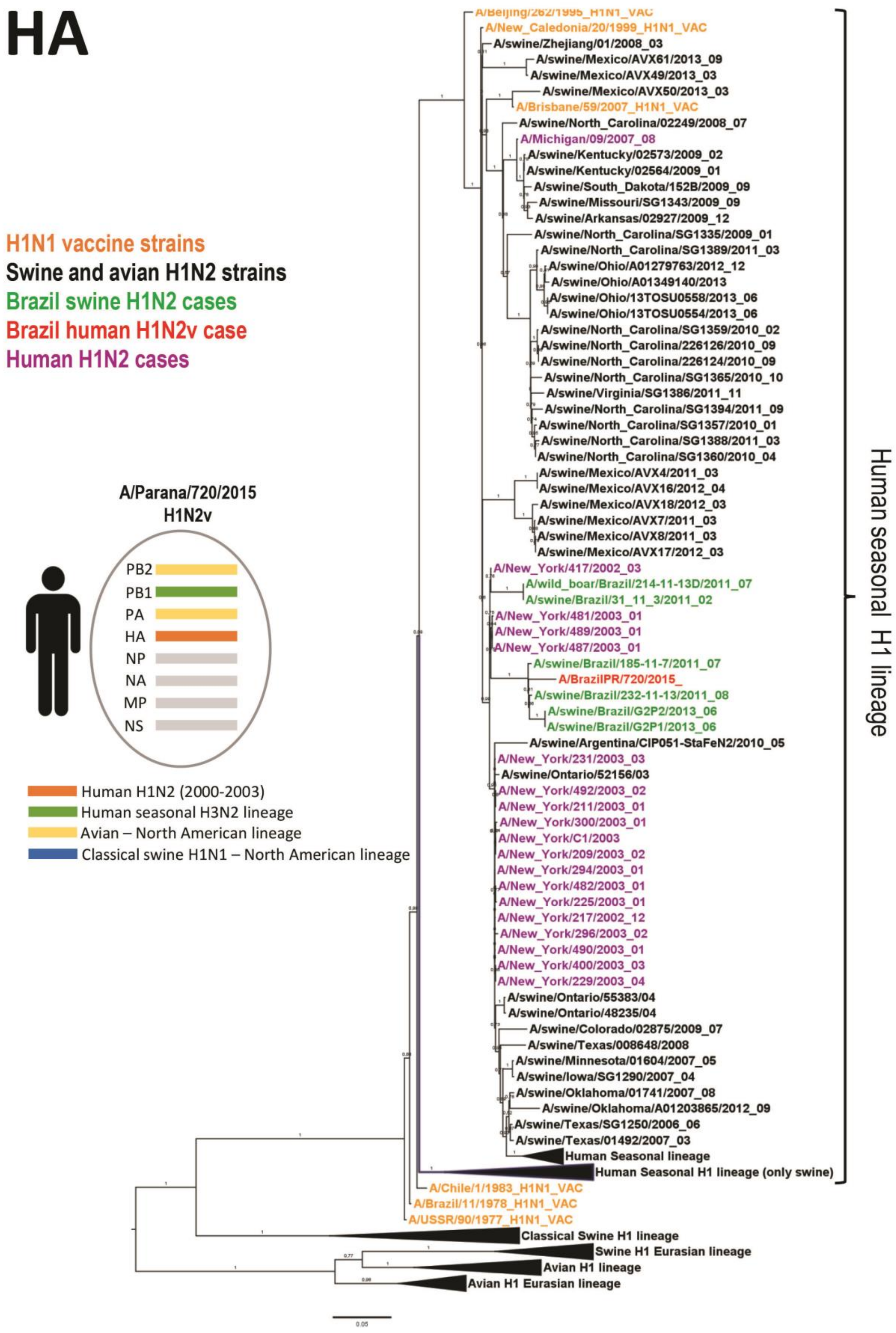
HA
H1N1 vaccine strains
Swine and avian H1N2 strains
Brazil swine H1N2 cases
Brazil human H1N2v case
Human H1N2 cases
A/Parana/720/2015
H1N2v
PB2
PB1
PA
HA
NP
NA
MP
NS
Human H1N2 (2000-2003)
Human seasonal H3N2 lineage
Avian – North American lineage
Classical swine H1N1 – North American lineage
A/Beijing/262/1995_H1N1_VAC
A/New_Caledonia/20/1999_H1N1_VAC
A/swine/Zhejiang/01/2008_03
A/swine/Mexico/AVX61/2013_09
A/swine/Mexico/AVX49/2013_03
A/swine/Mexico/AVX50/2013_03
A/Brisbane/59/2007_H1N1_VAC
A/swine/North_Carolina/02249/2008_07
A/Michigan/09/2007_08
A/swine/Kentucky/02573/2009_02
A/swine/Kentucky/02564/2009_01
A/swine/South_Dakota/152B/2009_09
A/swine/Missouri/SG1343/2009_09
A/swine/Arkansas/02927/2009_12
A/swine/North_Carolina/SG1335/2009_01
A/swine/North_Carolina/SG1389/2011_03
A/swine/Ohio/A01279763/2012_12
A/swine/Ohio/A01349140/2013
A/swine/Ohio/13TOSU0558/2013_06
A/swine/Ohio/13TOSU0554/2013_06
A/swine/North_Carolina/SG1359/2010_02
A/swine/North_Carolina/226126/2010_09
A/swine/North_Carolina/226124/2010_09
A/swine/North_Carolina/SG1365/2010_10
A/swine/Virginia/SG1386/2011_11
A/swine/North_Carolina/SG1394/2011_09
A/swine/North_Carolina/SG1357/2010_01
A/swine/North_Carolina/SG1388/2011_03
A/swine/North_Carolina/SG1360/2010_04
A/swine/Mexico/AVX4/2011_03
A/swine/Mexico/AVX16/2012_04
A/swine/Mexico/AVX18/2012_03
A/swine/Mexico/AVX7/2011_03
A/swine/Mexico/AVX8/2011_03
A/swine/Mexico/AVX17/2012_03
A/New_York/417/2002_03
A/wild_boar/Brazil/214-11-13D/2011_07
A/swine/Brazil/31_11_3/2011_02
A/New_York/481/2003_01
A/New_York/489/2003_01
A/New_York/487/2003_01
A/swine/Brazil/185-11-7/2011_07
A/BrazilPR/720/2015_
A/swine/Brazil/232-11-13/2011_08
A/swine/Brazil/G2P2/2013_06
A/swine/Brazil/G2P1/2013_06
A/swine/Argentina/CIP051-StaFeN2/2010_05
A/New_York/231/2003_03
A/swine/Ontario/52156/03
A/New_York/492/2003_02
A/New_York/211/2003_01
A/New_York/300/2003_01
A/New_York/C1/2003
A/New_York/209/2003_02
A/New_York/294/2003_01
A/New_York/482/2003_01
A/New_York/225/2003_01
A/New_York/217/2002_12
A/New_York/296/2003_02
A/New_York/490/2003_01
A/New_York/400/2003_03
A/New_York/229/2003_04
A/swine/Ontario/55383/04
A/swine/Ontario/48235/04
A/swine/Colorado/02875/2009_07
A/swine/Texas/008648/2008
A/swine/Minnesota/01604/2007_05
A/swine/Iowa/SG1290/2007_04
A/swine/Oklahoma/01741/2007_08
A/swine/Oklahoma/A01203865/2012_09
A/swine/Texas/SG1250/2006_06
A/swine/Texas/01492/2007_03
Human Seasonal lineage
Human Seasonal H1 lineage (only swine)
A/Chile/1/1983_H1N1_VAC
A/Brazil/11/1978_H1N1_VAC
A/USSR/90/1977_H1N1_VAC
Classical Swine H1 lineage
Swine H1 Eurasian lineage
Avian H1 lineage
Avian H1 Eurasian lineage
Human seasonal H1 lineage
0.05

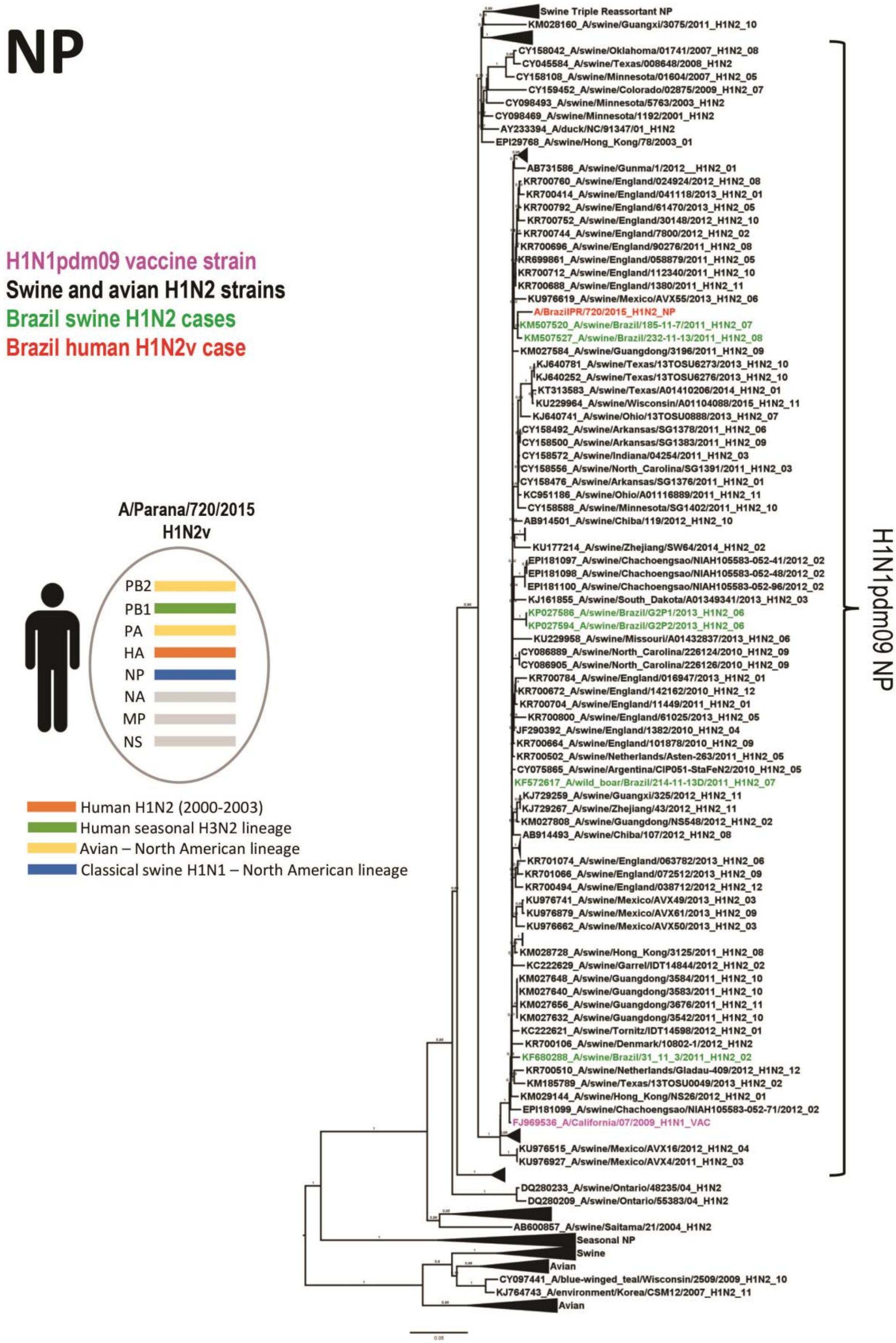
NP
H1N1pdm09 vaccine strain
Swine and avian H1N2 strains
Brazil swine H1N2 cases
Brazil human H1N2v case
A/Parana/720/2015
H1N2v
PB2
PB1
PA
HA
NP
NA
MP
NS
Human H1N2 (2000-2003)
Human seasonal H3N2 lineage
Avian – North American lineage
Classical swine H1N1 – North American lineage
Swine Triple Reassortant NP
KM028160_A/swine/Guangxi/3075/2011_H1N2_10
CY158042_A/swine/Oklahoma/01741/2007_H1N2_08
CY045584_A/swine/Texas/008648/2008_H1N2
CY158108_A/swine/Minnesota/01604/2007_H1N2_05
CY159452_A/swine/Colorado/02875/2009_H1N2_07
CY098493_A/swine/Minnesota/5763/2003_H1N2
CY098469_A/swine/Minnesota/1192/2001_H1N2
AY233394_A/duck/NC/91347/01_H1N2
EPI29768_A/swine/Hong_Kong/78/2003_01
AB731586_A/swine/Gunma/1/2012__H1N2_01
KR700760_A/swine/England/024924/2012_H1N2_08
KR700414_A/swine/England/041118/2013_H1N2_01
KR700792_A/swine/England/61470/2013_H1N2_05
KR700752_A/swine/England/30148/2012_H1N2_10
KR700744_A/swine/England/7800/2012_H1N2_02
KR700696_A/swine/England/90276/2011_H1N2_08
KR699861_A/swine/England/058879/2011_H1N2_05
KR700712_A/swine/England/112340/2011_H1N2_10
KR700688_A/swine/England/1380/2011_H1N2_11
KU976619_A/swine/Mexico/AVX55/2013_H1N2_06
A/BrazilPR/720/2015_H1N2_NP
KM507520_A/swine/Brazil/185-11-7/2011_H1N2_07
KM507527_A/swine/Brazil/232-11-13/2011_H1N2_08
KM027584_A/swine/Guangdong/3196/2011_H1N2_09
KJ640781_A/swine/Texas/13TOSU6273/2013_H1N2_10
KJ640252_A/swine/Texas/13TOSU6276/2013_H1N2_10
KT313583_A/swine/Texas/A01410206/2014_H1N2_01
KU229964_A/swine/Wisconsin/A01104088/2015_H1N2_11
KJ640741_A/swine/Ohio/13TOSU0888/2013_H1N2_07
CY158492_A/swine/Arkansas/SG1378/2011_H1N2_06
CY158500_A/swine/Arkansas/SG1383/2011_H1N2_09
CY158572_A/swine/Indiana/04254/2011_H1N2_03
CY158556_A/swine/North_Carolina/SG1391/2011_H1N2_03
CY158476_A/swine/Arkansas/SG1376/2011_H1N2_01
KC951186_A/swine/Ohio/A01116889/2011_H1N2_11
CY158588_A/swine/Minnesota/SG1402/2011_H1N2_10
AB914501_A/swine/Chiba/119/2012_H1N2_10
KU177214_A/swine/Zhejiang/SW64/2014_H1N2_02
EPI181097_A/swine/Chachoengsao/NIAH105583-052-41/2012_02
EPI181098_A/swine/Chachoengsao/NIAH105583-052-48/2012_02
EPI181100_A/swine/Chachoengsao/NIAH105583-052-96/2012_02
KJ161855_A/swine/South_Dakota/A01349341/2013_H1N2_03
KP027586_A/swine/Brazil/G2P1/2013_H1N2_06
KP027594_A/swine/Brazil/G2P2/2013_H1N2_06
KU229958_A/swine/Missouri/A01432837/2013_H1N2_06
CY086889_A/swine/North_Carolina/226124/2010_H1N2_09
CY086905_A/swine/North_Carolina/226126/2010_H1N2_09
KR700784_A/swine/England/016947/2013_H1N2_01
KR700672_A/swine/England/142162/2010_H1N2_12
KR700704_A/swine/England/11449/2011_H1N2_01
KR700800_A/swine/England/61025/2013_H1N2_05
JF290392_A/swine/England/1382/2010_H1N2_04
KR700664_A/swine/England/101878/2010_H1N2_09
KR700502_A/swine/Netherlands/Asten-263/2011_H1N2_05
CY075865_A/swine/Argentina/CIP051-StaFeN2/2010_H1N2_05
KF572617_A/wild_boar/Brazil/214-11-13D/2011_H1N2_07
KJ729259_A/swine/Guangxi/325/2012_H1N2_11
KJ729267_A/swine/Zhejiang/43/2012_H1N2_11
KM027808_A/swine/Guangdong/NS548/2012_H1N2_02
AB914493_A/swine/Chiba/107/2012_H1N2_08
KR701074_A/swine/England/063782/2013_H1N2_06
KR701066_A/swine/England/072512/2013_H1N2_09
KR700494_A/swine/England/038712/2012_H1N2_12
KU976741_A/swine/Mexico/AVX49/2013_H1N2_03
KU976879_A/swine/Mexico/AVX61/2013_H1N2_09
KU976662_A/swine/Mexico/AVX50/2013_H1N2_03
KM028728_A/swine/Hong_Kong/3125/2011_H1N2_08
KC222629_A/swine/Garrel/IDT14844/2012_H1N2_02
KM027648_A/swine/Guangdong/3584/2011_H1N2_10
KM027640_A/swine/Guangdong/3583/2011_H1N2_10
KM027656_A/swine/Guangdong/3676/2011_H1N2_11
KM027632_A/swine/Guangdong/3542/2011_H1N2_10
KC222621_A/swine/Tornitz/IDT14598/2012_H1N2_01
KR700106_A/swine/Denmark/10802-1/2012_H1N2
KF680288_A/swine/Brazil/31_11_3/2011_H1N2_02
KR700510_A/swine/Netherlands/Gladau-409/2012_H1N2_12
KM185789_A/swine/Texas/13TOSU0049/2013_H1N2_02
KM029144_A/swine/Hong_Kong/NS26/2012_H1N2_01
EPI181099_A/swine/Chachoengsao/NIAH105583-052-71/2012_02
FJ969536_A/California/07/2009_H1N1_VAC
KU976515_A/swine/Mexico/AVX16/2012_H1N2_04
KU976927_A/swine/Mexico/AVX4/2011_H1N2_03
H1N1pdm09 NP
DQ280233_A/swine/Ontario/48235/04_H1N2
DQ280209_A/swine/Ontario/55383/04_H1N2
AB600857_A/swine/Saitama/21/2004_H1N2
Seasonal NP
Swine
Avian
CY097441_A/blue-winged_teal/Wisconsin/2509/2009_H1N2_10
KJ764743_A/environment/Korea/CSM12/2007_H1N2_11
Avian
0.05

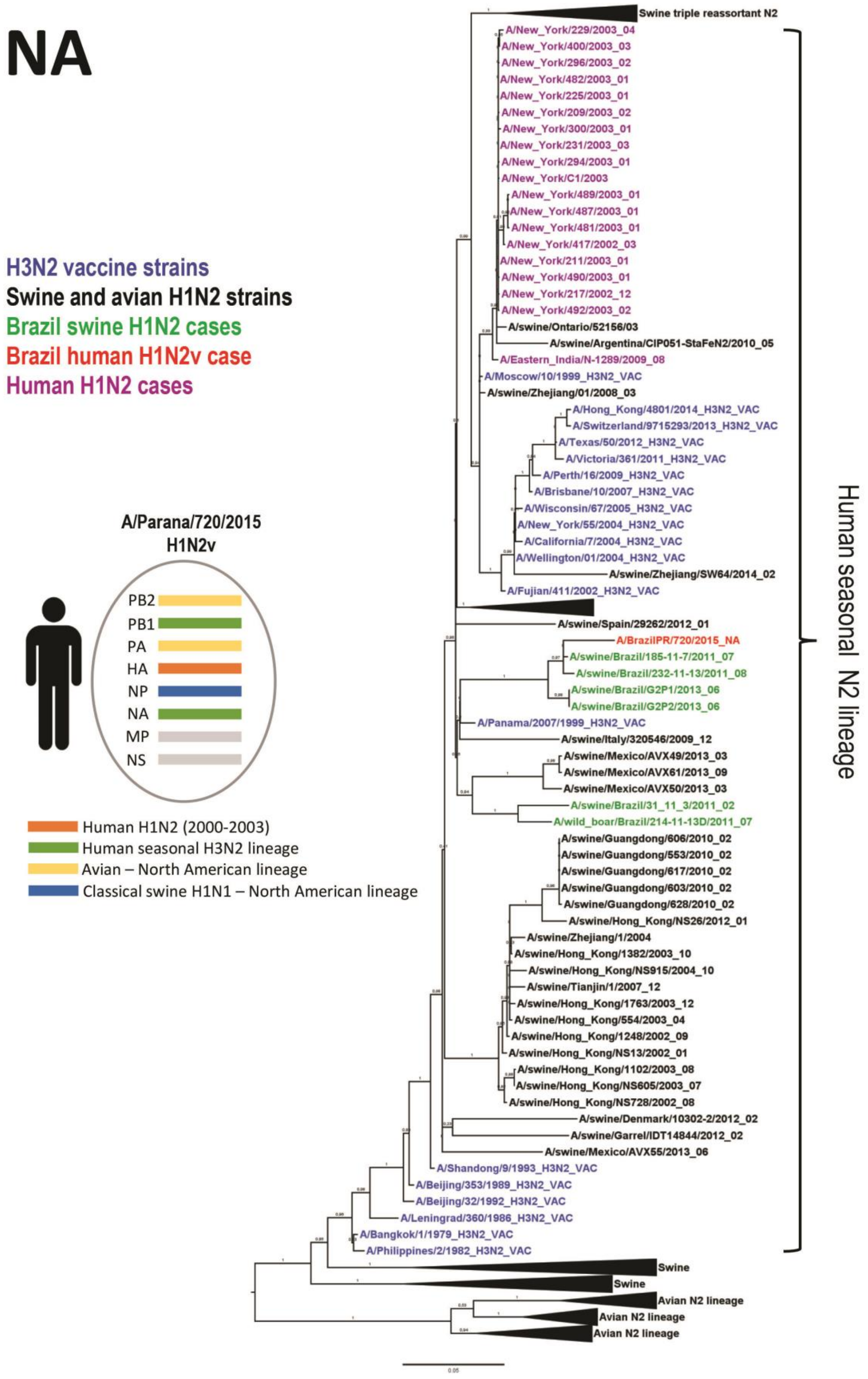
NA
H3N2 vaccine strains
Swine and avian H1N2 strains
Brazil swine H1N2 cases
Brazil human H1N2v case
Human H1N2 cases
A/Parana/720/2015
H1N2v
PB2
PB1
PA
HA
NP
NA
MP
NS
Human H1N2 (2000-2003)
Human seasonal H3N2 lineage
Avian – North American lineage
Classical swine H1N1 – North American lineage
Swine triple reassortant N2
A/New_York/229/2003_04
A/New_York/400/2003_03
A/New_York/296/2003_02
A/New_York/482/2003_01
A/New_York/225/2003_01
A/New_York/209/2003_02
A/New_York/300/2003_01
A/New_York/231/2003_03
A/New_York/294/2003_01
A/New_York/C1/2003
A/New_York/489/2003_01
A/New_York/487/2003_01
A/New_York/481/2003_01
A/New_York/417/2002_03
A/New_York/211/2003_01
A/New_York/490/2003_01
A/New_York/217/2002_12
A/New_York/492/2003_02
A/swine/Ontario/52156/03
A/swine/Argentina/CIP051-StaFeN2/2010_05
A/Eastern_India/N-1289/2009_08
A/Moscow/10/1999_H3N2_VAC
A/swine/Zhejiang/01/2008_03
A/Hong_Kong/4801/2014_H3N2_VAC
A/Switzerland/9715293/2013_H3N2_VAC
A/Texas/50/2012_H3N2_VAC
A/Victoria/361/2011_H3N2_VAC
A/Perth/16/2009_H3N2_VAC
A/Brisbane/10/2007_H3N2_VAC
A/Wisconsin/67/2005_H3N2_VAC
A/New_York/55/2004_H3N2_VAC
A/California/7/2004_H3N2_VAC
A/Wellington/01/2004_H3N2_VAC
A/swine/Zhejiang/SW64/2014_02
A/Fujian/411/2002_H3N2_VAC
A/swine/Spain/29262/2012_01
A/BrazilPR/720/2015_NA
A/swine/Brazil/185-11-7/2011_07
A/swine/Brazil/232-11-13/2011_08
A/swine/Brazil/G2P1/2013_06
A/swine/Brazil/G2P2/2013_06
A/Panama/2007/1999_H3N2_VAC
A/swine/Italy/320546/2009_12
A/swine/Mexico/AVX49/2013_03
A/swine/Mexico/AVX61/2013_09
A/swine/Mexico/AVX50/2013_03
A/swine/Brazil/31_11_3/2011_02
A/wild_boar/Brazil/214-11-13D/2011_07
A/swine/Guangdong/606/2010_02
A/swine/Guangdong/553/2010_02
A/swine/Guangdong/617/2010_02
A/swine/Guangdong/603/2010_02
A/swine/Guangdong/628/2010_02
A/swine/Hong_Kong/NS26/2012_01
A/swine/Zhejiang/1/2004
A/swine/Hong_Kong/1382/2003_10
A/swine/Hong_Kong/NS915/2004_10
A/swine/Tianjin/1/2007_12
A/swine/Hong_Kong/1763/2003_12
A/swine/Hong_Kong/554/2003_04
A/swine/Hong_Kong/1248/2002_09
A/swine/Hong_Kong/NS13/2002_01
A/swine/Hong_Kong/1102/2003_08
A/swine/Hong_Kong/NS605/2003_07
A/swine/Hong_Kong/NS728/2002_08
A/swine/Denmark/10302-2/2012_02
A/swine/Garrel/IDT14844/2012_02
A/swine/Mexico/AVX55/2013_06
A/Shandong/9/1993_H3N2_VAC
A/Beijing/353/1989_H3N2_VAC
A/Beijing/32/1992_H3N2_VAC
A/Leningrad/360/1986_H3N2_VAC
A/Bangkok/1/1979_H3N2_VAC
A/Philippines/2/1982_H3N2_VAC
Swine
Swine
Avian N2 lineage
Avian N2 lineage
Avian N2 lineage
Human seasonal N2 lineage

MP

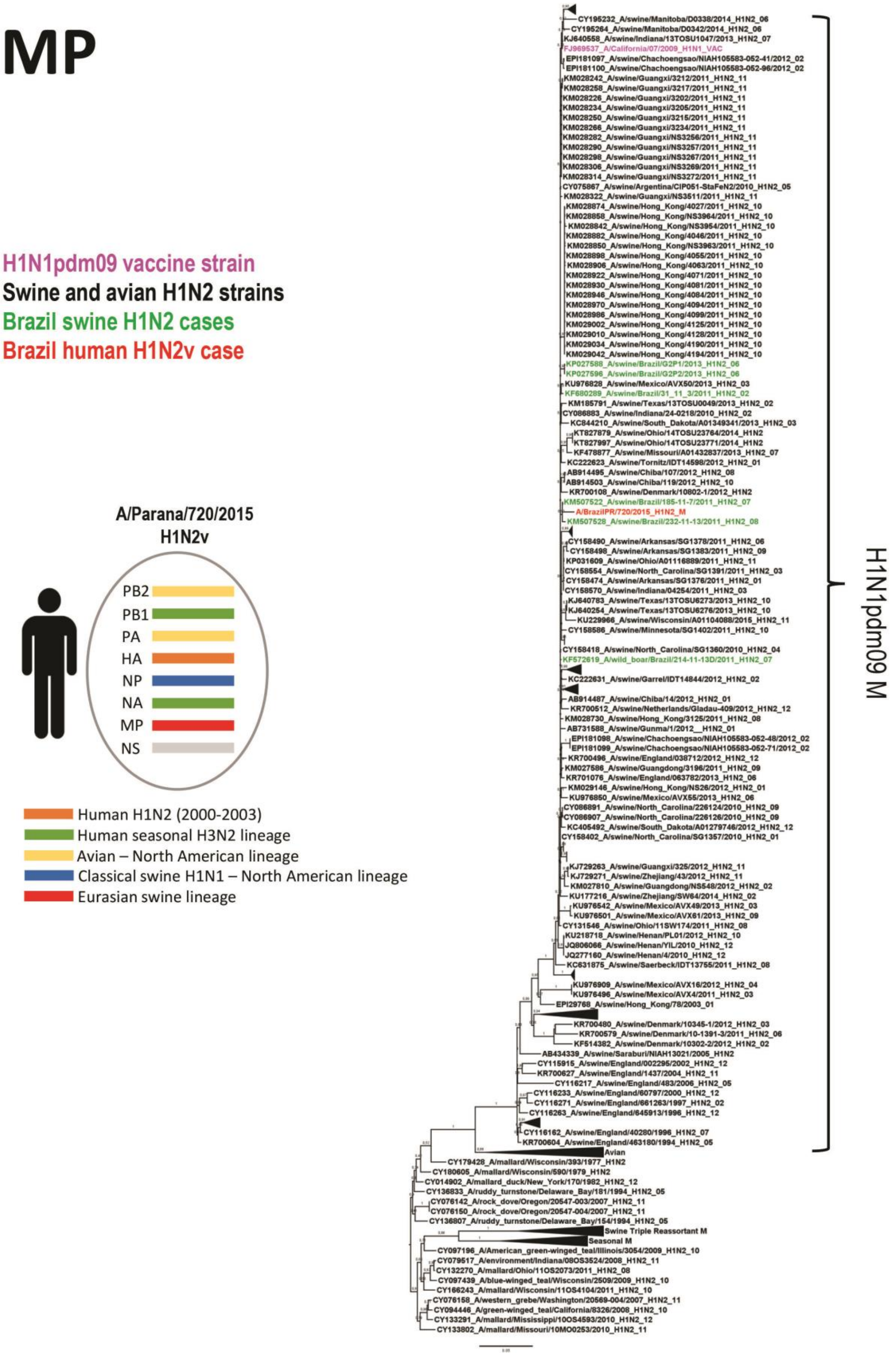
H1N1pdm09 vaccine strain
Swine and avian H1N2 strains
Brazil swine H1N2 cases
Brazil human H1N2v case
A/Parana/720/2015
H1N2v
PB2
PB1
PA
HA
NP
NA
MP
NS
Human H1N2 (2000-2003)
Human seasonal H3N2 lineage
Avian – North American lineage
Classical swine H1N1 – North American lineage
Eurasian swine lineage
H1N1pdm09 M
FJ969537_A/California/07/2009_H1N1_VAC
KP027588_A/swine/Brazil/G2P1/2013_H1N2_06
KP027596_A/swine/Brazil/G2P2/2013_H1N2_06
KF680289_A/swine/Brazil/31_11_3/2011_H1N2_02
KM507522_A/swine/Brazil/185-11-7/2011_H1N2_07
A/BrazilPR/720/2015_H1N2_M
KM507528_A/swine/Brazil/232-11-13/2011_H1N2_08
KF572619_A/wild_boar/Brazil/214-11-13D/2011_H1N2_07
Avian
Swine Triple Reassortant M
Seasonal M

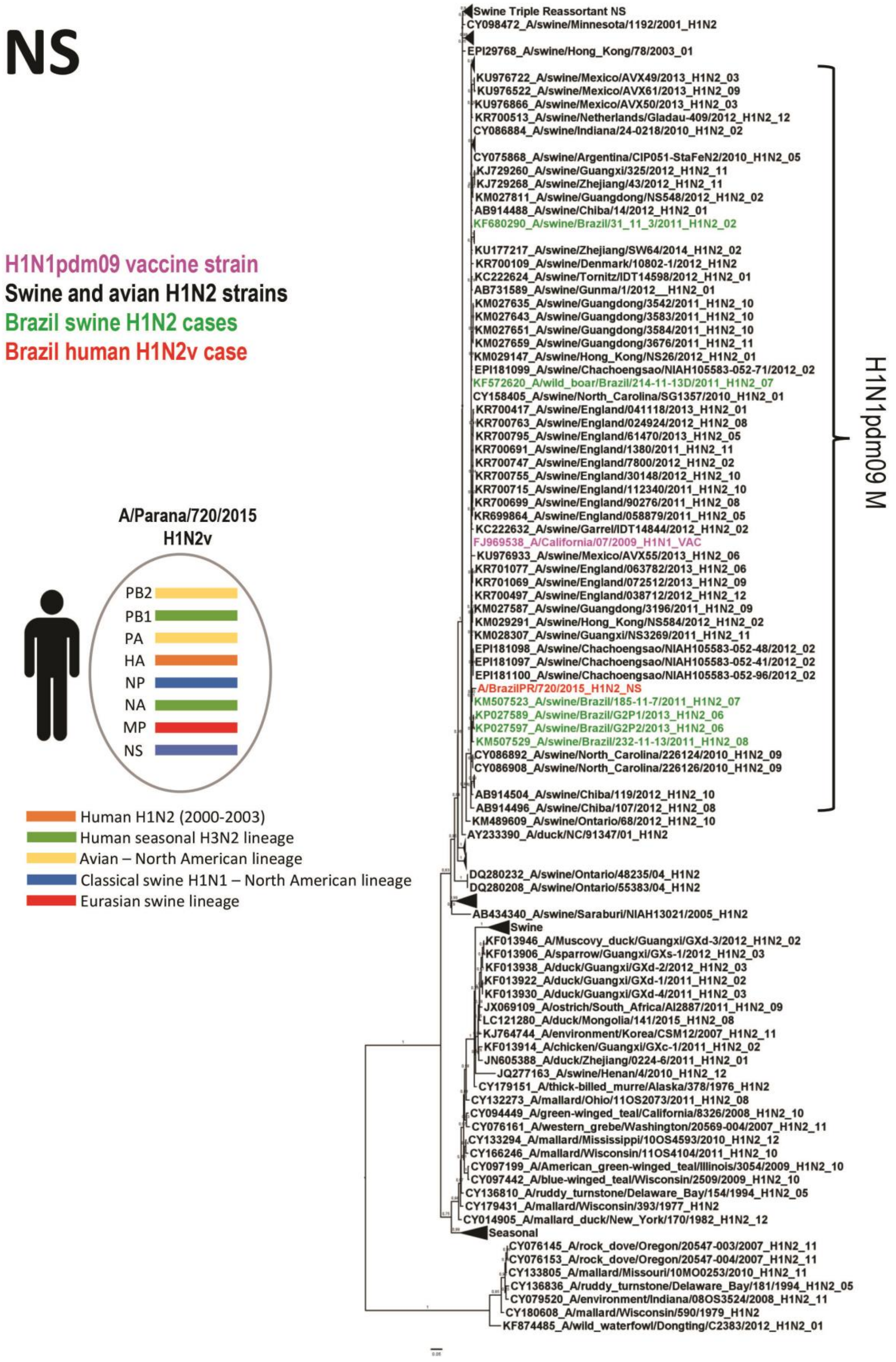

NS
H1N1pdm09 vaccine strain
Swine and avian H1N2 strains
Brazil swine H1N2 cases
Brazil human H1N2v case
A/Parana/720/2015
H1N2v
PB2
PB1
PA
HA
NP
NA
MP
NS
Human H1N2 (2000-2003)
Human seasonal H3N2 lineage
Avian – North American lineage
Classical swine H1N1 – North American lineage
Eurasian swine lineage
Swine Triple Reassortant NS
CY098472_A/swine/Minnesota/1192/2001_H1N2
EPI29768_A/swine/Hong_Kong/78/2003_01
KU976722_A/swine/Mexico/AVX49/2013_H1N2_03
KU976522_A/swine/Mexico/AVX61/2013_H1N2_09
KU976866_A/swine/Mexico/AVX50/2013_H1N2_03
KR700513_A/swine/Netherlands/Gladau-409/2012_H1N2_12
CY086884_A/swine/Indiana/24-0218/2010_H1N2_02
CY075868_A/swine/Argentina/CIP051-StaFeN2/2010_H1N2_05
KJ729260_A/swine/Guangxi/325/2012_H1N2_11
KJ729268_A/swine/Zhejiang/43/2012_H1N2_11
KM027811_A/swine/Guangdong/NS548/2012_H1N2_02
AB914488_A/swine/Chiba/14/2012_H1N2_01
KF680290_A/swine/Brazil/31_11_3/2011_H1N2_02
KU177217_A/swine/Zhejiang/SW64/2014_H1N2_02
KR700109_A/swine/Denmark/10802-1/2012_H1N2
KC222624_A/swine/Tornitz/IDT14598/2012_H1N2_01
AB731589_A/swine/Gunma/1/2012__H1N2_01
KM027635_A/swine/Guangdong/3542/2011_H1N2_10
KM027643_A/swine/Guangdong/3583/2011_H1N2_10
KM027651_A/swine/Guangdong/3584/2011_H1N2_10
KM027659_A/swine/Guangdong/3676/2011_H1N2_11
KM029147_A/swine/Hong_Kong/NS26/2012_H1N2_01
EPI181099_A/swine/Chachoengsao/NIAH105583-052-71/2012_02
KF572620_A/wild_boar/Brazil/214-11-13D/2011_H1N2_07
CY158405_A/swine/North_Carolina/SG1357/2010_H1N2_01
KR700417_A/swine/England/041118/2013_H1N2_01
KR700763_A/swine/England/024924/2012_H1N2_08
KR700795_A/swine/England/61470/2013_H1N2_05
KR700691_A/swine/England/1380/2011_H1N2_11
KR700747_A/swine/England/7800/2012_H1N2_02
KR700755_A/swine/England/30148/2012_H1N2_10
KR700715_A/swine/England/112340/2011_H1N2_10
KR700699_A/swine/England/90276/2011_H1N2_08
KR699864_A/swine/England/058879/2011_H1N2_05
KC222632_A/swine/Garrel/IDT14844/2012_H1N2_02
FJ969538_A/California/07/2009_H1N1_VAC
KU976933_A/swine/Mexico/AVX55/2013_H1N2_06
KR701077_A/swine/England/063782/2013_H1N2_06
KR701069_A/swine/England/072512/2013_H1N2_09
KR700497_A/swine/England/038712/2012_H1N2_12
KM027587_A/swine/Guangdong/3196/2011_H1N2_09
KM029291_A/swine/Hong_Kong/NS584/2012_H1N2_02
KM028307_A/swine/Guangxi/NS3269/2011_H1N2_11
EPI181098_A/swine/Chachoengsao/NIAH105583-052-48/2012_02
EPI181097_A/swine/Chachoengsao/NIAH105583-052-41/2012_02
EPI181100_A/swine/Chachoengsao/NIAH105583-052-96/2012_02
A/BrazilPR/720/2015_H1N2_NS
KM507523_A/swine/Brazil/185-11-7/2011_H1N2_07
KP027589_A/swine/Brazil/G2P1/2013_H1N2_06
KP027597_A/swine/Brazil/G2P2/2013_H1N2_06
KM507529_A/swine/Brazil/232-11-13/2011_H1N2_08
CY086892_A/swine/North_Carolina/226124/2010_H1N2_09
CY086908_A/swine/North_Carolina/226126/2010_H1N2_09
AB914504_A/swine/Chiba/119/2012_H1N2_10
AB914496_A/swine/Chiba/107/2012_H1N2_08
KM489609_A/swine/Ontario/68/2012_H1N2_10
H1N1pdm09 M
AY233390_A/duck/NC/91347/01_H1N2
DQ280232_A/swine/Ontario/48235/04_H1N2
DQ280208_A/swine/Ontario/55383/04_H1N2
AB434340_A/swine/Saraburi/NIAH13021/2005_H1N2
Swine
KF013946_A/Muscovy_duck/Guangxi/GXd-3/2012_H1N2_02
KF013906_A/sparrow/Guangxi/GXs-1/2012_H1N2_03
KF013938_A/duck/Guangxi/GXd-2/2012_H1N2_03
KF013922_A/duck/Guangxi/GXd-1/2011_H1N2_02
KF013930_A/duck/Guangxi/GXd-4/2011_H1N2_03
JX069109_A/ostrich/South_Africa/AI2887/2011_H1N2_09
LC121280_A/duck/Mongolia/141/2015_H1N2_08
KJ764744_A/environment/Korea/CSM12/2007_H1N2_11
KF013914_A/chicken/Guangxi/GXc-1/2011_H1N2_02
JN605388_A/duck/Zhejiang/0224-6/2011_H1N2_01
JQ277163_A/swine/Henan/4/2010_H1N2_12
CY179151_A/thick-billed_murre/Alaska/378/1976_H1N2
CY132273_A/mallard/Ohio/11OS2073/2011_H1N2_08
CY094449_A/green-winged_teal/California/8326/2008_H1N2_10
CY076161_A/western_grebe/Washington/20569-004/2007_H1N2_11
CY133294_A/mallard/Mississippi/10OS4593/2010_H1N2_12
CY166246_A/mallard/Wisconsin/11OS4104/2011_H1N2_10
CY097199_A/American_green-winged_teal/Illinois/3054/2009_H1N2_10
CY097442_A/blue-winged_teal/Wisconsin/2509/2009_H1N2_10
CY136810_A/ruddy_turnstone/Delaware_Bay/154/1994_H1N2_05
CY179431_A/mallard/Wisconsin/393/1977_H1N2
CY014905_A/mallard_duck/New_York/170/1982_H1N2_12
Seasonal
CY076145_A/rock_dove/Oregon/20547-003/2007_H1N2_11
CY076153_A/rock_dove/Oregon/20547-004/2007_H1N2_11
CY133805_A/mallard/Missouri/10MO0253/2010_H1N2_11
CY136836_A/ruddy_turnstone/Delaware_Bay/181/1994_H1N2_05
CY079520_A/environment/Indiana/08OS3524/2008_H1N2_11
CY180608_A/mallard/Wisconsin/590/1979_H1N2
KF874485_A/wild_waterfowl/Dongting/C2383/2012_H1N2_01
